# 4-Nitro­isophthalic acid

**DOI:** 10.1107/S1600536811053797

**Published:** 2011-12-21

**Authors:** Yang-Hui Luo, Mei-Ling Pan

**Affiliations:** a Key Laboratory of Urban and Architectural Heritage Conservation, (Southeast University), Ministry of Education, Nanjing 210096, People’s Republic of China, and College of Chemistry and Chemical Engineering, Southeast University, Nanjing 210096, People’s Republic of China

## Abstract

In the crystal structure of the title compound, C_8_H_5_NO_6_, both carboxyl groups are involved in inter­molecular centrosymmetric cyclic O—H⋯O hydrogen-bonding associations, which give a zigzag chain structure extending along (2
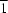
1). Weak π–π stacking inter­actions are also present [minimum ring centroid separation = 3.893 (4) Å].

## Related literature

For 4-nitro­isophthalic acid as an inter­mediate in the synthesis of pharmaceutical drugs and as a ligand in transition metal complexes, see: Birk & Weihe (2009 ▶); Pan *et al.* (2011 ▶).
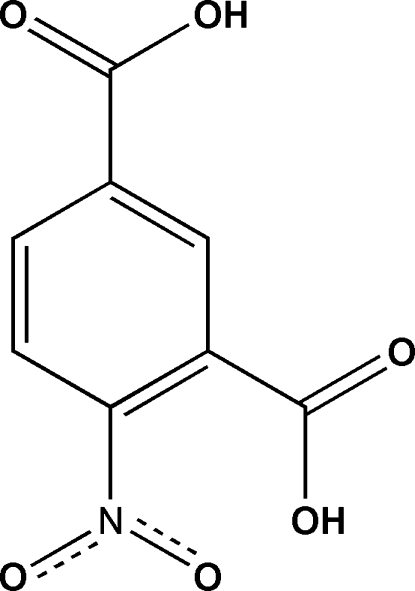


## Experimental

### 

#### Crystal data

C_8_H_5_NO_6_
*M* *_r_* = 211.13
Triclinic, 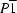

*a* = 7.0261 (14) Å
*b* = 7.4380 (15) Å
*c* = 8.5775 (17) Å
α = 80.09 (3)°
β = 86.22 (3)°
γ = 75.37 (3)°
*V* = 427.14 (15) Å^3^
*Z* = 2
Mo *K*α radiation
μ = 0.15 mm^−1^
*T* = 293 K
0.30 × 0.25 × 0.20 mm

#### Data collection

Rigaku SCXmini CCD-detector diffractometer
Absorption correction: multi-scan (*CrystalClear*; Rigaku, 2005 ▶) *T*_min_ = 0.957, *T*_max_ = 0.971
4103 measured reflections
1943 independent reflections
1554 reflections with *I* > 2σ(*I*)
*R*_int_ = 0.136

#### Refinement

*R*[*F* ^2^ > 2σ(*F* ^2^)] = 0.092
*wR*(*F* ^2^) = 0.308
*S* = 0.86
1943 reflections
136 parameters
8 restraints
H-atom parameters constrained
Δρ_max_ = 0.40 e Å^−3^
Δρ_min_ = −0.50 e Å^−3^

### 

Data collection: *CrystalClear* (Rigaku, 2005 ▶); cell refinement: *CrystalClear*; data reduction: *CrystalClear*; program(s) used to solve structure: *SHELXS97* (Sheldrick, 2008 ▶); program(s) used to refine structure: *SHELXL97* (Sheldrick, 2008 ▶); molecular graphics: *DIAMOND* (Brandenburg & Putz, 2005 ▶); software used to prepare material for publication: *SHELXL97*.

## Supplementary Material

Crystal structure: contains datablock(s) I, New_Global_Publ_Block. DOI: 10.1107/S1600536811053797/zs2171sup1.cif

Structure factors: contains datablock(s) I. DOI: 10.1107/S1600536811053797/zs2171Isup2.hkl

Supplementary material file. DOI: 10.1107/S1600536811053797/zs2171Isup3.cml

Additional supplementary materials:  crystallographic information; 3D view; checkCIF report

## Figures and Tables

**Table 1:** Hydrogen-bond geometry (Å, °)

*D*—H⋯*A*	*D*—H	H⋯*A*	*D*⋯*A*	*D*—H⋯*A*
O4—H4⋯O3^i^	0.86	1.76	2.605 (7)	168
O5—H5⋯O6^ii^	0.87	1.73	2.602 (7)	180

Symmetry codes: (i) 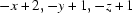; (ii) 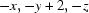.

**Fig. 1.**
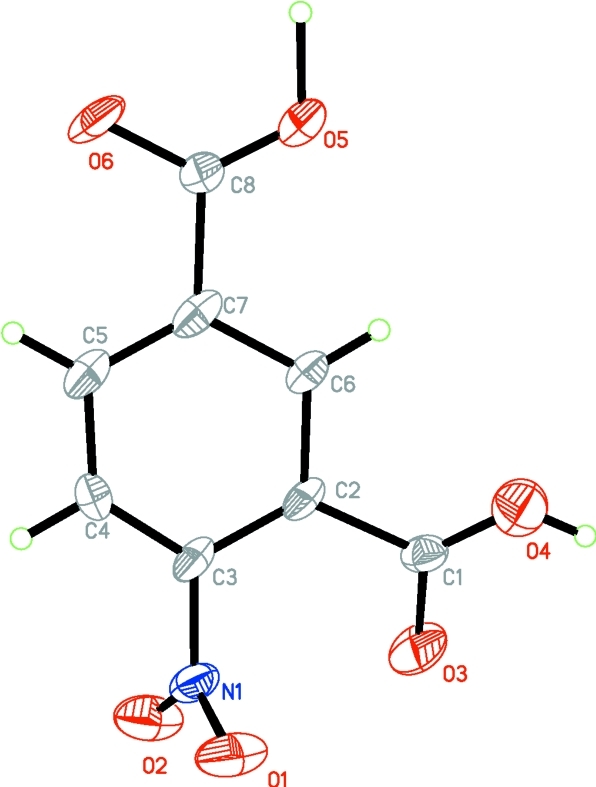
The molecular structure of the title compound with the atom-labelling scheme. Displacement ellipsoids are drawn at the 30% probability level.

**Fig. 2.**
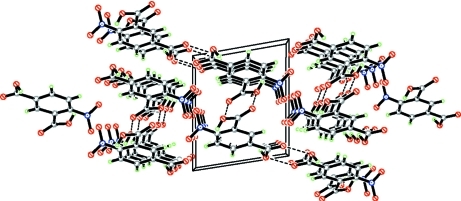
A packing diagram viewed down the a axis showing the three-dimensional network. Intermolecular hydrogen bonds are shown as dashed lines.

## supplementary crystallographic information

### Comment

4-Nitroisophthalic acid is an important chemical material because it is an intermediate in the synthesis of many pharmaceutical drugs and is also an excellent ligand for many transition metal complexes (Pan*et al.*, 2011; Birk & Weihe, 2009). As part of our interest in this compound, we report here the crystal structure of this acid.

The molecular structure of the title compound, C_8_H_5_NO_6_ is shown in Fig. 1. All of the non-H and non-O atoms are approximately coplanar: the maximu r.m.s. deviation being 0.0202 Å. In the crystal structure, both carboxylic acid groups are involved in intermolecular centrosymmetric cyclic O—H···O hydrogen-bonding associations (Table 1) which give a zigzag chain structure extending along (2 -1 1) (Fig. 2). Weak π···π stacking interactions are also present [minimum ring centroid separation = 3.893 (4) Å].

### Experimental

4-Nitroisophthalic acid was obtained commercially from ChemFuture PharmaTech, Ltd (Nanjing, Jiangsu). Crystals of it suitable for X-ray diffraction were obstained by slow evaporation of a methanol solution.

### Refinement

All H atoms attached to C atoms and O atoms were fixed geometrically and treated as riding with C—H = 0.93 Å, and O—H = 0.86±1 Å with *U*_iso_(H) = 1.2*U*_eq_(C).

### Crystal data

**Table:**

C_8_H_5_NO_6_	*Z* = 2
*M**_r_* = 211.13	*F*(000) = 216
Triclinic, *P*1	*D*_x_ = 1.642 Mg m^−^^3^
Hall symbol: -P 1	Mo *K*α radiation, λ = 0.71073 Å
*a* = 7.0261 (14) Å	Cell parameters from 1943 reflections
*b* = 7.4380 (15) Å	θ = 3.0–27.5°
*c* = 8.5775 (17) Å	µ = 0.15 mm^−^^1^
α = 80.09 (3)°	*T* = 293 K
β = 86.22 (3)°	Prism, red
γ = 75.37 (3)°	0.30 × 0.25 × 0.20 mm
*V* = 427.14 (15) Å^3^	

### Data collection

**Table:**

Rigaku SCXmini CCD-detector diffractometer	1943 independent reflections
Radiation source: fine-focus sealed tube	1554 reflections with *I* > 2σ(*I*)
graphite	*R*_int_ = 0.136
Detector resolution: 13.6612 pixels mm^-1^	θ_max_ = 27.5°, θ_min_ = 3.0°
CCD profile–fitting scans	*h* = −9→9
Absorption correction: multi-scan *CrystalClear* (Rigaku, 2005)	*k* = −9→9
*T*_min_ = 0.957, *T*_max_ = 0.971	*l* = −11→11
4103 measured reflections	

### Refinement

**Table:**

Refinement on *F*^2^	Primary atom site location: structure-invariant direct methods
Least-squares matrix: full	Secondary atom site location: difference Fourier map
*R*[*F*^2^ > 2σ(*F*^2^)] = 0.092	Hydrogen site location: inferred from neighbouring sites
*wR*(*F*^2^) = 0.308	H-atom parameters constrained
*S* = 0.86	*w* = 1/[σ^2^(*F*_o_^2^) + (0.1225*P*)^2^] where *P* = (*F*_o_^2^ + 2*F*_c_^2^)/3
1943 reflections	(Δ/σ)_max_ = 0.017
136 parameters	Δρ_max_ = 0.40 e Å^−^^3^
8 restraints	Δρ_min_ = −0.50 e Å^−^^3^

### Special details

**Table:**

Geometry. Bond distances, angles etc. have been calculated using the rounded fractional coordinates. All su's are estimated from the variances of the (full) variance-covariance matrix. The cell esds are taken into account in the estimation of distances, angles and torsion angles
Refinement. Refinement of *F*^2^ against ALL reflections. The weighted *R*-factor *wR* and goodness of fit *S* are based on *F*^2^, conventional *R*-factors *R* are based on *F*, with *F* set to zero for negative *F*^2^. The threshold expression of *F*^2^ > σ(*F*^2^) is used only for calculating *R*-factors(gt) *etc*. and is not relevant to the choice of reflections for refinement. *R*-factors based on *F*^2^ are statistically about twice as large as those based on *F*, and *R*- factors based on ALL data will be even larger.

### Fractional atomic coordinates and isotropic or equivalent isotropic displacement parameters (Å^2^)

**Table:**

	*x*	*y*	*z*	*U*_iso_*/*U*_eq_	
O1	0.7042 (7)	0.0658 (7)	0.5109 (6)	0.0760 (19)	
O2	0.8052 (7)	−0.0122 (8)	0.2874 (6)	0.081 (2)	
O3	0.9385 (6)	0.3293 (8)	0.4061 (6)	0.076 (2)	
O4	0.7716 (6)	0.6167 (8)	0.4485 (6)	0.074 (2)	
O5	0.2226 (6)	0.9309 (7)	0.0974 (5)	0.0637 (18)	
O6	0.0288 (6)	0.7626 (7)	0.0224 (6)	0.0706 (18)	
N1	0.7089 (7)	0.1015 (8)	0.3680 (6)	0.0470 (19)	
C1	0.7884 (8)	0.4702 (9)	0.3938 (7)	0.041 (2)	
C2	0.6144 (7)	0.4407 (9)	0.3070 (6)	0.0396 (19)	
C3	0.5813 (8)	0.2668 (9)	0.2931 (7)	0.044 (2)	
C4	0.4265 (8)	0.2656 (9)	0.2113 (7)	0.0446 (19)	
C5	0.2929 (8)	0.4233 (10)	0.1433 (7)	0.049 (2)	
C6	0.4838 (7)	0.6123 (9)	0.2397 (6)	0.0397 (19)	
C7	0.3223 (7)	0.5954 (10)	0.1615 (6)	0.045 (2)	
C8	0.1809 (8)	0.7812 (9)	0.0882 (7)	0.045 (2)	
H4	0.87560	0.63520	0.48410	0.0880*	
H4A	0.40800	0.14920	0.19970	0.0540*	
H5	0.13830	1.03360	0.05700	0.0770*	
H5A	0.18720	0.41500	0.08750	0.0590*	
H6A	0.50480	0.72900	0.24730	0.0480*	

### Atomic displacement parameters (Å^2^)

**Table:**

	*U*^11^	*U*^22^	*U*^33^	*U*^12^	*U*^13^	*U*^23^
O1	0.075 (3)	0.068 (4)	0.066 (3)	0.011 (3)	−0.018 (3)	0.005 (3)
O2	0.098 (4)	0.058 (4)	0.067 (3)	0.017 (3)	−0.008 (3)	−0.006 (3)
O3	0.034 (2)	0.080 (4)	0.105 (4)	0.007 (3)	−0.030 (2)	−0.010 (3)
O4	0.045 (2)	0.097 (4)	0.085 (4)	−0.009 (3)	−0.029 (2)	−0.032 (3)
O5	0.049 (2)	0.067 (4)	0.074 (3)	−0.006 (3)	−0.034 (2)	−0.008 (3)
O6	0.042 (2)	0.086 (4)	0.079 (3)	−0.001 (3)	−0.034 (2)	−0.010 (3)
N1	0.046 (3)	0.053 (4)	0.036 (3)	−0.006 (3)	−0.015 (2)	0.005 (3)
C1	0.035 (3)	0.047 (4)	0.041 (4)	−0.012 (3)	−0.007 (3)	−0.001 (3)
C2	0.025 (3)	0.052 (4)	0.038 (3)	0.000 (3)	−0.012 (2)	−0.007 (3)
C3	0.032 (3)	0.055 (4)	0.043 (4)	−0.002 (3)	−0.012 (3)	−0.012 (3)
C4	0.048 (3)	0.032 (3)	0.060 (4)	−0.017 (3)	−0.011 (3)	−0.010 (3)
C5	0.038 (3)	0.067 (5)	0.044 (4)	−0.010 (4)	−0.016 (3)	−0.011 (3)
C6	0.032 (3)	0.052 (4)	0.036 (3)	−0.011 (3)	−0.012 (2)	−0.004 (3)
C7	0.030 (3)	0.071 (5)	0.032 (3)	−0.004 (3)	−0.009 (2)	−0.010 (3)
C8	0.037 (3)	0.046 (4)	0.054 (4)	−0.006 (3)	−0.013 (3)	−0.012 (3)

### Geometric parameters (Å, °)

**Table:**

O1—N1	1.209 (7)	C2—C6	1.424 (9)
O2—N1	1.225 (8)	C2—C3	1.396 (9)
O3—C1	1.281 (8)	C3—C4	1.335 (8)
O4—C1	1.235 (9)	C4—C5	1.370 (9)
O5—C8	1.237 (8)	C5—C7	1.383 (10)
O6—C8	1.289 (7)	C6—C7	1.398 (7)
O4—H4	0.8600	C7—C8	1.543 (9)
O5—H5	0.8700	C4—H4A	0.9300
N1—C3	1.406 (8)	C5—H5A	0.9300
C1—C2	1.552 (8)	C6—H6A	0.9300
			
C1—O4—H4	118.00	C4—C5—C7	117.0 (5)
C8—O5—H5	116.00	C2—C6—C7	116.2 (6)
O1—N1—C3	118.7 (5)	C5—C7—C8	120.9 (5)
O2—N1—C3	119.2 (5)	C6—C7—C8	116.3 (6)
O1—N1—O2	121.5 (6)	C5—C7—C6	122.7 (6)
O4—C1—C2	120.2 (6)	O6—C8—C7	115.3 (5)
O3—C1—O4	126.0 (6)	O5—C8—O6	126.5 (6)
O3—C1—C2	113.8 (5)	O5—C8—C7	118.2 (5)
C3—C2—C6	121.0 (5)	C3—C4—H4A	118.00
C1—C2—C6	113.4 (5)	C5—C4—H4A	118.00
C1—C2—C3	125.6 (5)	C4—C5—H5A	122.00
N1—C3—C4	122.9 (6)	C7—C5—H5A	121.00
N1—C3—C2	118.9 (5)	C2—C6—H6A	122.00
C2—C3—C4	118.2 (6)	C7—C6—H6A	122.00
C3—C4—C5	124.8 (6)		
			
O1—N1—C3—C2	69.8 (8)	C3—C2—C6—C7	0.6 (8)
O1—N1—C3—C4	−108.8 (7)	N1—C3—C4—C5	176.5 (6)
O2—N1—C3—C2	−118.3 (6)	C2—C3—C4—C5	−2.1 (9)
O2—N1—C3—C4	63.1 (8)	C3—C4—C5—C7	0.0 (9)
O3—C1—C2—C3	23.2 (8)	C4—C5—C7—C6	2.5 (8)
O3—C1—C2—C6	−157.0 (5)	C4—C5—C7—C8	179.5 (5)
O4—C1—C2—C3	−154.2 (6)	C2—C6—C7—C5	−2.7 (8)
O4—C1—C2—C6	25.5 (8)	C2—C6—C7—C8	−179.9 (5)
C1—C2—C3—N1	2.8 (9)	C5—C7—C8—O5	−174.4 (5)
C1—C2—C3—C4	−178.6 (5)	C5—C7—C8—O6	5.5 (8)
C6—C2—C3—N1	−177.0 (5)	C6—C7—C8—O5	2.8 (8)
C6—C2—C3—C4	1.7 (8)	C6—C7—C8—O6	−177.3 (5)
C1—C2—C6—C7	−179.2 (5)		

### Hydrogen-bond geometry (Å, °)

**Table:**

*D*—H···*A*	*D*—H	H···*A*	*D*···*A*	*D*—H···*A*
O4—H4···O3^i^	0.86	1.76	2.605 (7)	168
O5—H5···O6^ii^	0.87	1.73	2.602 (7)	180
C5—H5A···O6^iii^	0.93	2.56	3.423 (8)	154

Symmetry codes: (i) −*x*+2, −*y*+1, −*z*+1; (ii) −*x*, −*y*+2, −*z*; (iii) −*x*, −*y*+1, −*z*.

## References

[bb1] Birk, T. & Weihe, H. (2009). *J. Chem. Crystallogr.* **39**, 766–771.

[bb2] Brandenburg, K. & Putz, H. (2005). *DIAMOND* Crystal Impact GbR, Bonn, Germany.

[bb3] Pan, M.-L., Luo, Y.-H. & Mao, S.-L. (2011). *Acta Cryst.* E**67**, o2345. 10.1107/S1600536811031837 PMC3200684 22065729

[bb4] Rigaku. (2005). *CrystalClear* Rigaku Corporation, Tokyo, Japan.

[bb5] Sheldrick, G. M. (2008). *Acta Cryst.* A**64**, 112–122. 10.1107/S0108767307043930 18156677

